# Transient peripheral edema following displaced corneal graft after descemet stripping automated endothelial keratoplasty (DSAEK): case presentation

**DOI:** 10.1186/1471-2415-11-37

**Published:** 2011-12-07

**Authors:** George D Kymionis, Dimitra M Portaliou, Takeshi Ide, Sonia H Yoo

**Affiliations:** 1 Institute of Vision and Optics University of Crete, Greece; 2 Bascom Palmer Eye Institute, University of Miami, Florida, USA

## Abstract

**Background:**

Descemet's Stripping with Automated Endothelial Keratoplasty (DSAEK) is constantly gaining popularity in the management of endothelial dysfunctions such as bullous keratopathy or Fuchs' dystrophy.

**Case Presentation:**

A 36 year - old man with Fuchs' dystrophy underwent combined phacoemulsification and DSAEK of the right eye. Immediately postoperatively, corneal graft displacement and peripheral corneal edema which remained stable during the first postoperative month were evident on slit lamp examination. Three months after the procedure the peripheral edema had completely resolved and the patients' subjective symptoms were improved.

**Conclusions:**

The purpose of this case presentation is to demonstrate that corneal graft displacement after DSAEK can lead to peripheral corneal edema that can resolve without further intervention such as graft repositioning or replacement.

## Background

Endothelial dysfunction and secondary corneal edema were traditionally considered indications for penetrating keratoplasty (PKP). Nevertheless, the array of post - PKP complications such as suture - induced irregular astigmatism, delayed visual rehabilitation and high graft rejection rate have led to the development and increasing popularity of techniques that selectively transplant the endothelium [1].

Descemet's Stripping with Automated Endothelial Keratoplasty (DSAEK) [2-4] is a procedure designed to replace diseased corneal endothelium with a healthy donor graft. In DSAEK, the donor lamellar graft is created by a microkeratome which minimizes stromal irregularities and increases visual rehabilitation and recovery.

In this case report, we present a patient with transient peripheral corneal edema following corneal graft displacement after DSAEK.

## Case Report

A 36 year - old man was referred to our institute with complaints about pain in both eyes, blurred vision especially on awakening and bilateral progressive visual loss, worse in the left eye. His uncorrected distance visual acuity (UDVA) was 20/40 in the right eye and 20/30 in the left eye. Corrected distance visual acuity (CDVA) was 20/32 in both eyes. The manifest refraction was -0.75/+0.50 × 135 for the right eye and -0.25 for the left eye respectively.

Slit lamp examination revealed bilateral corneal stromal edema and corneal guttata consistent with Fuchs' endothelial dystrophy.

Six months after the initial diagnosis, the patient underwent combined phacoemulsification with intraocular lens implantation followed by DSAEK on the right eye. The donor's age was 52 years while endothelial cell density (ECD) of the graft was 2619 cells/mm^2^. The surgical procedure was performed using the technique previously published by Terry MA et al [5] with the exception of not scraping the peripheral recipient stromal bed. An 8.25 mm folded donor lenticule was inserted with a Charlie forceps (Bauch & Lomb, Bausch & Lomb World Headquarters, One Bausch & Lomb Place Rochester, NY 14604-2701) through a 5 mm incision and tamponaded with an intracameral air bubble for 10 minutes after wound closure with one 10-0 nylon suture. A 50% air bubble remained after air fluid exchange. Postoperative medications included moxifloxacin four times daily for a week and prednisolone acetate 1% four times daily as well as artificial tears at least six times per day for a month postoperatively.

**Figure 1:**
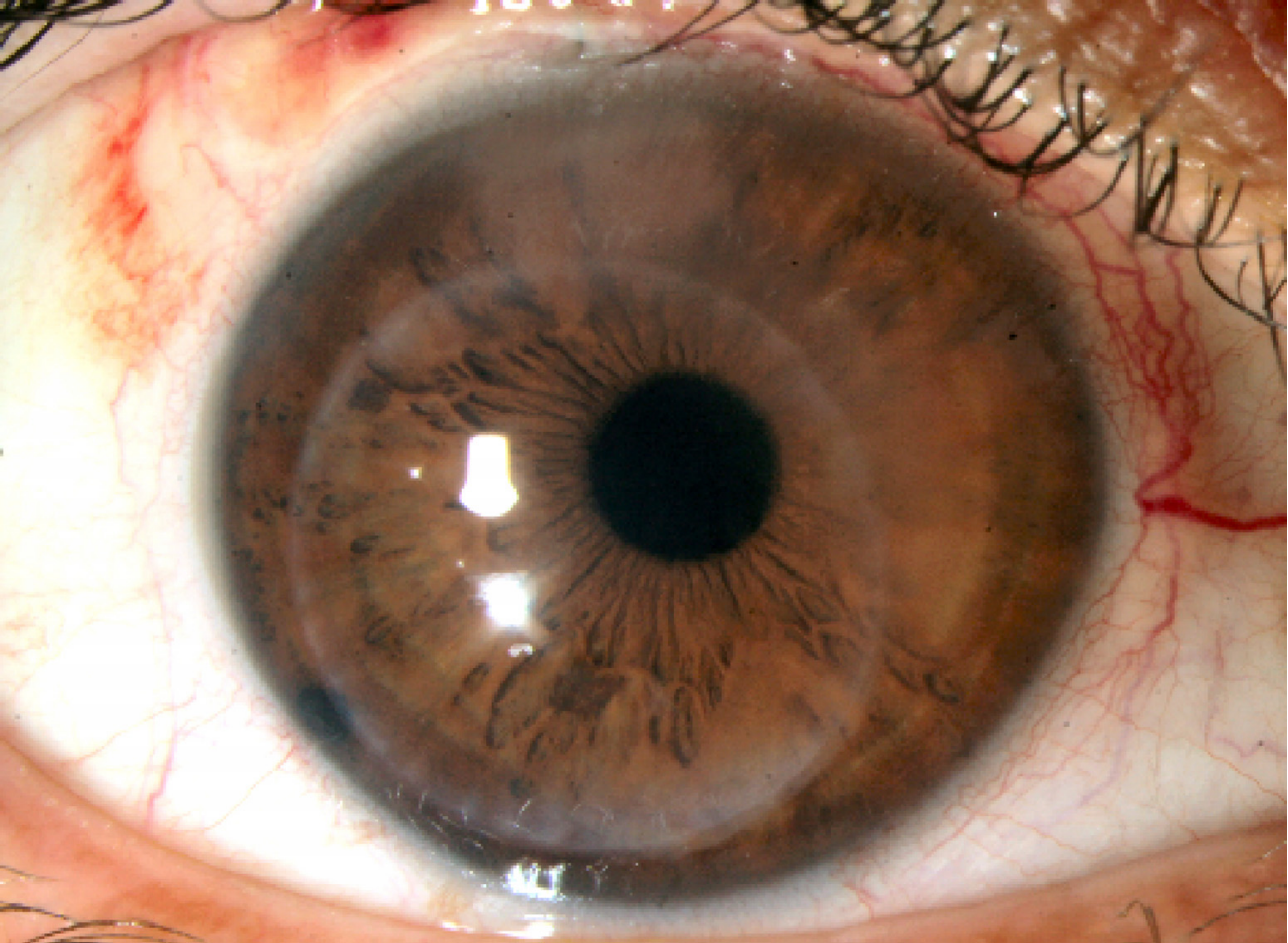
**Slit lamp photo at the first postoperative month**. Displaced corneal graft and superior nasal peripheral corneal edema are evident.

On postoperative day one, corneal graft displacement (eccentric button) without dislocation or interface fluid was seen on slit lamp examination. The central cornea gradually cleared during the first postoperative month while the peripheral superonasal corneal edema in the "uncovered" (by the displaced corneal graft) area remained (Figure 1). The patient had subjective symptoms due to the peripheral edema including peripheral blurred vision that was worse at night. Due to the patient's improved vision, donor graft replacement was not performed.

**Figure 2:**
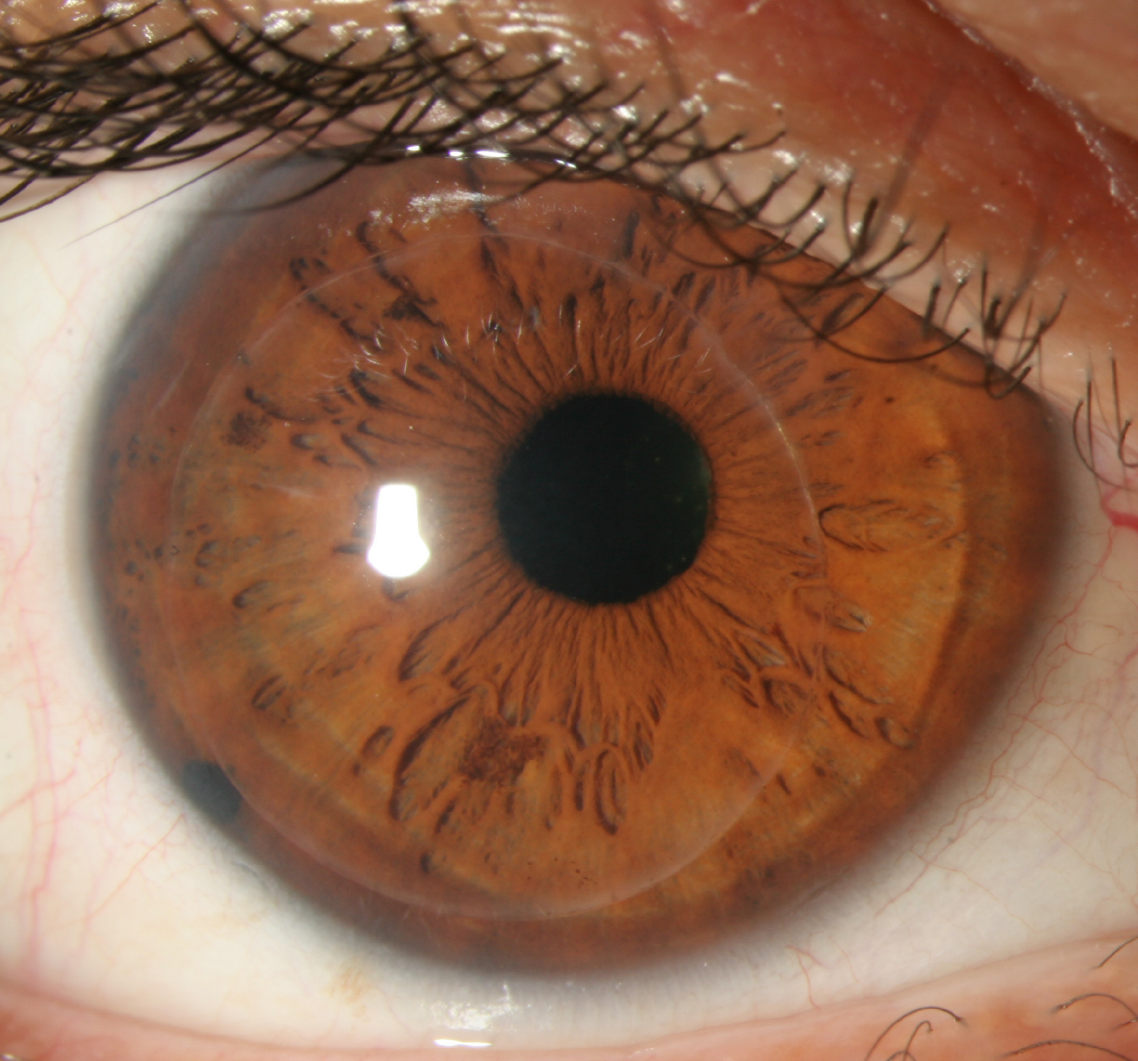
**Three months postoperatively, peripheral edema has receded**.

On the third postoperative month's visit, the cornea remained clear and the peripheral edema had completely resolved (Figure 2). The patient's UDVA was 20/40 while the CDVA was improved to 20/25 (+0.50/+0.50 × 175). Patients' initial corneal thickness in the area of the displaced graft was 743 μm and 3 months later decreased to 685 μm.

## Conclusions

Descemet's Stripping Automated Endothelial Keratoplasty (DSAEK) is a revolutionary surgical procedure that allows replacement of the diseased endothelium in cases of endothelial corneal dystrophies and bullous keratopathy. In such cases, PKP can be avoided and a less invasive approach can be employed.

One of the main concerns when performing DSAEK is donor graft adherence in the immediate postoperative period. It has been reported that 15 to 30% of the donor grafts are not fully attached and may be dislocated or floating in the anterior chamber (AC) [3,4]. Possible explanations for that could be presence of interface fluid or air, patient squeezing and eye rubbing, and problems with the preparation, handling, and insertion of lamellar donor tissue [6]. Additionally, the learning curve for the DSAEK technique, as it is a fairly new procedure, could account for graft detachments and dislocations.

Covert DJ et al [7] effectuated a combined procedure of phacoemulsification followed by DSAEK resulting in higher rates of graft detachment (3 out of 21 eyes) in respect to simple DSAEK procedures probably due to the need for further intraoperative manipulations.

Detachments or dislocations are usually treated with additional air injection into the AC or with graft exchange when adherence cannot be achieved.

In this case report we present a patient with displaced donor graft after DSAEK and associated peripheral corneal edema that resolved without any further intervention three months postoperatively.

It is assumed based on the patient's clinical outcome that even though the donor graft was displaced, the transplanted endothelial cells continued active pumping [7] of water and ions from the corneal stroma. Ultimately, stromal deturgescence and transparency [8] even in areas uncovered by the displaced donor graft were effectuated.

This phenomenon can be explained by presuming that the cornea is a structure that works as an integral. The functioning endothelial cells were sufficient enough to meet the needs of the cornea and to counteract pre-existing edema.

Other possible mechanisms that may have contributed to the eventual clearing of the peripheral corneal edema are endothelial cell migration to areas uncovered by the graft and/or dislocated endothelial cells function. Despite the clearing of the corneal edema, the edge of the donor graft being close to the photopic pupil margin would be expected to cause problems with visual quality such as night vision disturbances and increased higher order aberrations.

Similar cases of detached but not displaced grafts have been reported in the literature. Balachandran et al [9] described 2 cases of spontaneous corneal clearance after Descemet membrane endothelial keratoplasty (DMEK) suggesting endothelial transfer, migration, regeneration, or a combination thereof from either the donor or the recipient as possible explanations of the phenomenon. Dirisamer et al [10] analyzed the presence of different re- endothelialization patterns after DMEK such as massive endothelial migration or some form of cell signaling to draw donor endothelial cells toward the recipient posterior stroma ("homing").

In conclusion, displaced corneal graft after DSAEK can lead to transient corneal edema in areas uncovered by a displaced graft which can be resolved within a few months without the need of further intervention such as donor button replacement or repositioning.

## Consent

Written informed consent was obtained from the patient for publication of this case report and any accompanying images. A copy of the written consent is available for review by the Editor-in-Chief of this journal.

## Competing interests

The authors declare that they have no competing interests.

## Authors' contributions

GDK conceived of the case report and participated in its design and coordination, DMP performed manuscript writing, TI helped to draft the manuscript and SHY performed the surgery. All authors read and approved the final manuscript.

## Funding

The authors have no financial or proprietary interest in any materials or methods described herein.

## Pre-publication history

The pre-publication history for this paper can be accessed here:

http://www.biomedcentral.com/1471-2415/11/37/prepub
